# Molecular Systematic of Three Species of *Oithona* (Copepoda, Cyclopoida) from the Atlantic Ocean: Comparative Analysis Using 28S rDNA

**DOI:** 10.1371/journal.pone.0035861

**Published:** 2012-04-27

**Authors:** Georgina D. Cepeda, Leocadio Blanco-Bercial, Ann Bucklin, Corina M. Berón, María D. Viñas

**Affiliations:** 1 Instituto Nacional de Investigación y Desarrollo Pesquero (INIDEP), Mar del Plata, Argentina; 2 Centro de Estudios de Biodiversidad y Biotecnología (CEBB-CIB-FIBA), Mar del Plata, Argentina; 3 Consejo Nacional de Investigaciones Científicas y Técnicas (CONICET), Buenos Aires, Argentina; 4 Instituto de Investigaciones Marinas y Costeras (IIMyC), Facultad de Ciencias Exactas y Naturales, Universidad Nacional de Mar del Plata, Consejo Nacional de Investigaciones Científicas y Técnicas, Mar del Plata, Argentina; 5 Department of Marine Sciences, University of Connecticut, Groton, Connecticut, United States of America; Biodiversity Insitute of Ontario - University of Guelph, Canada

## Abstract

Species of *Oithona* (Copepoda, Cyclopoida) are highly abundant, ecologically important, and widely distributed throughout the world oceans. Although there are valid and detailed descriptions of the species, routine species identifications remain challenging due to their small size, subtle morphological diagnostic traits, and the description of geographic forms or varieties. This study examined three species of *Oithona* (*O. similis*, *O. atlantica* and *O. nana*) occurring in the Argentine sector of the South Atlantic Ocean based on DNA sequence variation of a 575 base-pair region of 28S rDNA, with comparative analysis of these species from other North and South Atlantic regions. DNA sequence variation clearly resolved and discriminated the species, and revealed low levels of intraspecific variation among North and South Atlantic populations of each species. The 28S rDNA region was thus shown to provide an accurate and reliable means of identifying the species throughout the sampled domain. Analysis of 28S rDNA variation for additional species collected throughout the global ocean will be useful to accurately characterize biogeographical distributions of the species and to examine phylogenetic relationships among them.

## Introduction

### Biogeography and Ecology of the Species

Among the small size copepods, the family Oithonidae [1] is recognized as one of the most abundant groups in the ocean [2]. The abundance, biomass and ecological role of *Oithona* spp. have been examined in recent studies [3]–[5]. The genus has been the subject of concerted and expert taxonomic analysis and detailed descriptions of the species are in place [6]–[8]. However, routine identification of species has remained challenging due to the small body size and subtle mophological differences among species [6] and descriptions of geographic forms or varieties of widely-distributed species [9].

The *Oithona* species examined in this study are important components of the Argentine Sea - a region of the Southwest Atlantic Ocean -, as well as of the North Atlantic Ocean [5], [10]–[12]. Over the Argentine continental shelf, the occurrence of *O. similis* Claus 1866 [13] syn. *O. helgolandica* [14], [15], *O. atlantica* [16] and *O. nana* [17] has been extensively cited [18]–[20]. These species are abundant, ecologically-important, and geographically-widespread; their numerical dominance was recently highlighted [21], [22]. *Oithona similis* occurs over the Argentine continental shelf between 34° and 55° S [18], [23]–[25]. It is broadly distributed from the tropics to high latitudes of the Atlantic [10], [18], [23]–[25] and Pacific Oceans [8]; in the Indian Ocean, and Mediterranean and Red Seas [26]. Although *O. similis* is a widespread species, multivariate analyses of community structure in the Argentine Sea reveal that the species reaches its maximum densities in cold shelf waters [20], [27].

*Oithona atlantica* also has a broad biogeographical distribution throughout both the North and South Atlantic Oceans, occurring over wide ranges in salinity (24–26 ppt and 34–36 ppt) and temperature (8–19°C) [18]. Despite such wide ecological tolerances, this is the least abundant *Oithona* species in the Argentine Sea [24], [28], but quite common throughout the Strait of Magellan [18]. It occurs in the northern North and eastern equatorial Pacific Ocean, Bearing Sea and Sea of Japan [8]. It is also found in the Sub-Antarctic and Antarctic waters, as well as the Mediterranean Sea [8].

In Argentine waters *O. nana* is found throughout the year between 34° and 45°****S. The species is an important component of the coastal species assemblage [27], [28], and it is potentially important as prey for fish larvae [29], [30]. It is also found in tropical and subtropical waters of the Atlantic Ocean [31], [32] as well as in the Mediterranean Sea [33], and the Pacific and Indian Oceans [8].

### 28S rDNA as a Taxonomic Marker

Although molecular approaches have been applied exhaustively to copepods to ensure accurate taxonomic identification of species, little information is available for cyclopoid copepods, especially for species of *Oithona*. DNA sequence variation of the large-subunit (28S) rRNA gene has been used extensively to examine phylogenetic relationships among marine invertebrate species, including cnidarians [34], annelids [35], nematodes [36], molluscs [37], and echinoderms [38], among others. The broad application of this gene as a character for taxonomic identification of species with subtle or ambiguous morphological characteristics makes it a useful marker to be employed for species of the cyclopoid copepod *Oithona*.

The relationships among *Oithona* species, including *O. similis, O. atlantica* and *O. nana*, have been studied for the Pacific and Indian Oceans [8]. These morphological analyses included forty five structural characters and suggested that *O. atlantica* and *O. similis* are more closely related to each other than to *O. nana* [8]. Here we analyze DNA sequences for a 575 base-pair (bp) region of the 28S rRNA gene and characterize patterns of variation within and among three *Oithona* species occurring in the South and North Atlantic Oceans.

## Methods

### Ethics Statement

No specific permits were required for the described field study, and no endangered or protected species were included in this study.

### Collection of Samples

**Figure 1:**
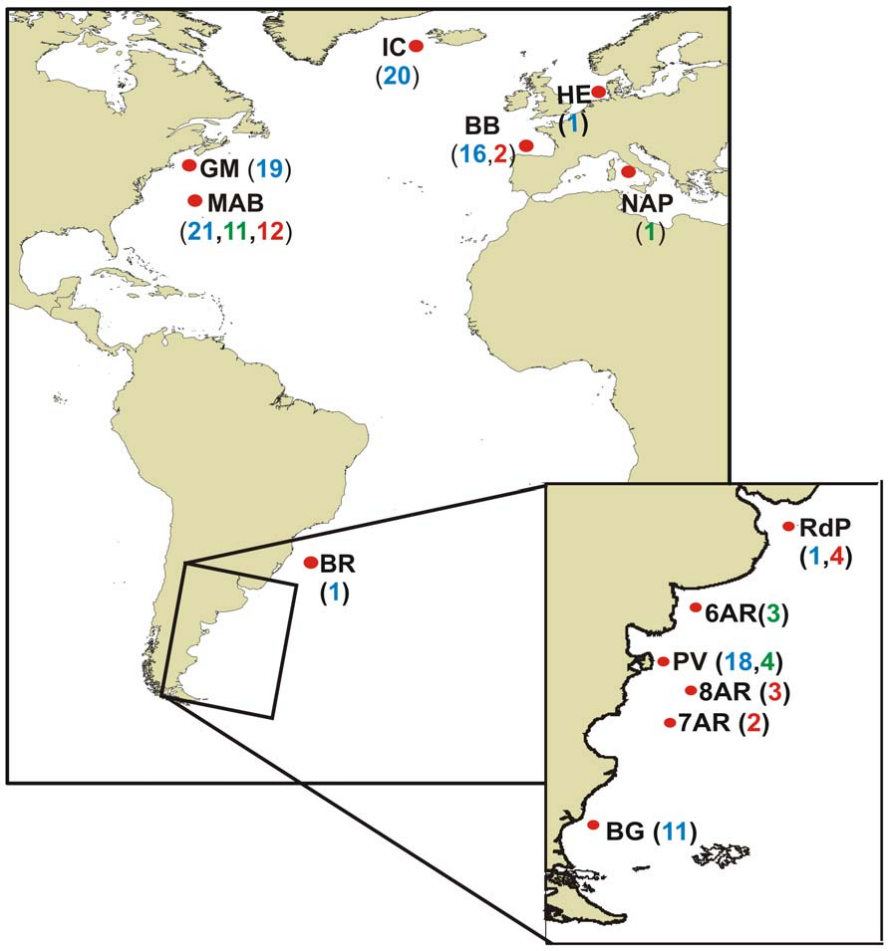
Collection sites and number of specimens in each site for each *Oithona* species. Specimens of *O. similis* sampled (in blue); *O. nana* (green); *O. atlantica* (red). Explanation of abbreviations for the collection sites are given in Table I and the text.

Zooplankton samples collected from regions across the North and South Atlantic Oceans (Figure 1, Table 1), preserved immediately and stored in 95% undenatured ethanol, as described by Bucklin [39]. A total of 150 oithonid copepods were identified to species level following [24], [25], using a Leica D1000 inverted microscope. The following specimens were removed and prepared for molecular analysis: *O. similis* (108 individuals), *O. nana* (19 individuals) and *O. atlantica* (23 individuals). Specimens from *O. similis* and *O. nana* type localities were also included in the molecular analysis.

**Table 1:** Sample sites, latitude, longitude, location code, sample size (N), sequence diversity (*h*), standarized sequence diversity (Hk) and number of kind sequences in each population of *O. similis*, *O. nana* and *O. atlantica* collected for this study from the Atlantic Ocean.

Species	Sample site	Latitude	Longitude	Location Code	N	h	H*k*
*O. similis*	Gulf of Maine, US	43°10′4.8″N	70°25′4.8″W	GM	19	0.51	0.52
	Bay of Biscay, Spain	43°42′N	6° 9′W	BB	16	0.24	0.24
	Iceland	64°20.15′N	27°W	IC	20	0.68	0.72
	Mid Atlantic Bight, US	38°16.3′N	74°24.4′W	MAB	21	0.74	0.72
	Península Valdés, Argentina	42°31′4.8″S	63°12′W	PV	18	0.81	0.82
	Bahía Grande, Argentina	51°S	67°W	BG	11	0.34	0.35
	Río de la Plata, Argentina	36°4′48″S	54°32′2.4″W	RdP	1	N/A	N/A
	Helgoland Sea, Germany*	54°10′57″N	7°53′E	HE	1	N/A	N/A
	Torres, Brazil	29°40′4.8″S	49°30′W	BR	1	N/A	N/A
					**108**		
*O. nana*	Mid Atlantic Bight, US	38°16.3′N	74°24.4′W	MAB	11	0.56	0.56
	El Rincón, Argentina	39°38′2.4″S	61°6′3.6″W	6AR	3	0.00	0.00
	Península Valdés, Argentina	42°31′4.8″S	63°12′W	PV	4	0.50	0.50
	Gulf of Naples, Italy*	40°50′N	14°15′E	NAP	1	N/A	N/A
					**19**		
*O. atlantica*	Bay of Biscay, Spain	43°42′N	6° 9′W	BB	2	1.00	1.00
	Mid Atlantic Bight, US	38°16.3′N	74°24.4′W	MAB	12	0.41	0.41
	Rio de la Plata, Argentina	36°4′48″S	54°32′2.4″W	RdP	4	0.00	0.00
	Argentina	45°15′S	62°30′3,6″W	7AR	2	0.00	0.00
	Argentina	43°31′4.8″S	61°23′2.4″W	8AR	3	0.00	0.00
					**23**		

Total sample size for each species is indicated in bold, samples from type locality are indicated by asterisk. N/A: not applicable.

### Molecular Analysis

DNA was extracted from individual identified specimens using the QIAGEN Dneasy tissue Kit. The Polymerase Chain Reaction (PCR) was used to amplify a 800 bp fragment of the D1–D2 region of the large subunit (28S) ribosomal DNA (rDNA) gene using primers 28SF1: 5′-GCGGAGGAAAAGAAACTAAC-3′ and 28SR1: 5′-GCATAGTTTCACCATCTTTCGGG-3′ [34]. PCR amplifications were performed in a total volume of 25 µl including 5 µl of 5X Green GoTaq® Flexi Buffer, 2.5 µl of 25 mM MgCl_2_, 1 µl of dNTPs (final concentration 0.2 mM each), 1 µl of each primer (10 µM), 0.75 units of GoTaq® Flexi DNA Polymerase (Promega) and 3 µl of the DNA template solution. The PCR protocol was: 4 min initial denaturation step at 94°C; 35 cycles of 40 s denaturation step at 94°C, 40 s annealing at 50°C, and 90 s extension at 72°C; and a final extension step of 15 min at 72°C.

Several sets of PCR primers for various genes were tested, but most did not amplified consistently. The genes for which published primers were tested included: internal transcribed spacer [40]; mitochondrial cytochrome c oxidase subunit I [41]; cytochrome *b* and 12S rDNA [42]; heat shock protein 70 [43]; and AMP-activated protein kinase [44].

Approximately 5 µl of each PCR product was electrophoresed on a 1% TBE agarose gel and visualized by UV light with with Biotium GelRed^TM^ staining. The PCR products were purified using QIAquick spin columns (Qiagen). Both strands of the template DNA were sequenced using the PCR primers and Big Dye Terminator Ver. 3.1 (Applied Biosystems Inc., ABI), and were run in an ABI 3130 Genetic Analyzer automated capillary DNA sequencer.

The 28S rDNA sequences obtained were manually edited, with comparison of aligned sequences for both strands. DNA sequences for *O. similis*, *O. nana* and *O. atlantica* were aligned using the default parameters by Clustal W [45], using MEGA Ver. 5.05 [46]. DNA sequences were submitted to the molecular database, GenBank (http://www.nlm.nih.ncbi.org) and were assigned a GenBank Accession Numbers: FM991727.1; JF419529-JF419547.

### Genetic Distances within and between Oithona Species

Analysis was done using a final aligned length of 575 bp of the 28S rRNA gene. Numbers of kind sequence and sequence diversities (*h*) were calculated for each population sampled for the studied species by DnaSP Ver. 5.10 [47]. Standarized sequence diversities (Hk) were calculated considering the smallest sample size (*O. similis*: n = 11; *O. nana*: n = 3; *O. atlantica:* n = 2) using the software RAREFAC (http://www.pierroton.inra.fr/genetics/labo/Software/Rarefac) [48]. The appropriate best-fit substitution model of DNA evolution was determined with jModelTest Ver. 0.1.1 [49] under the Akaike information criterion (A.I.C.). Neighbor-Joining method [50] analysis implemented in MEGA Ver 5.05 [46] was used on the identified kind sequences to assess the relationships among the three *Oithona* species based on DNA sequence variation; relative support for the tree topology was obtained by bootstrapping [51] using 10,000 iterations.

### Genetic Variation of O. similis

A total of 108 28S rDNA sequences for *O. similis* were aligned using MEGA Ver. 5.05 [46]. A 51-bp region showing intraspecific variation was used for this analysis; the best-fitting substitution model was determined with jModelTest [49]. The most appropriate model was found to Jukes-Cantor; the model and estimated parameters were set in Arlequin Ver. 3.5.1.2 [52] and the geographic pattern of 28S rDNA variation was assessed. Φ_ST_ genetic distances between all pairs of *O. similis* populations were calculated using Arlequin Ver. 3.5.1.2 [52]. Pairwise Φ_ST_ values among all conspecific populations were calculated and tested for significance through 10,000 permutations. For this analysis, all sequence types found in the populations from the Gulf of Maine (GM), Mid Atlantic Bight (MAB), Iceland (IC), Bay of Biscay (BB), Península Valdés (PV) and Bahía Grande (BG) were considered (Table 1).

An hierarchical Analysis of MOlecular VAriation [53] was performed using different groupings of populations based on the distances between sampling locations and Φ_ST_ distances. The statistical significance of the AMOVA statistics, including among groups (Φ_CT_), among populations within groups (Φ_SC_), and within populations (Φ_ST_), was obtained after 10,000 permutations.

## Results

### Genetic Distances within and between Oithona Species

DNA sequences of a 575 bp region of the 28S rDNA gene for 108 *O. similis* individuals revealed the presence of six well-resolved kind sequences and six kind sequences with one or two ambiguous sites (H1–H12). These ambigous sites correspoded to C-T sites, and were defined by equivalents peaks of both bases (Figure S1).

Among the 19 *O. nana* specimens from 3 populations, five kind sequences (H13–H17) defined by ten polymorphic sites were recorded, whereas among the 23 *O. atlantica* individuals analyzed, distributed in 5 populations, three kind sequences (H18–H20) were found defined by thirteen polymorphic sites. For *O. similis,* the sequence diversity was somewhat higher at PV than at MAB or IC. An intermediate value was found at GM, while the lower ones were at BG and BB (Table 1). For *O. nana*, mean values of sequence diversity were found at MAB and PV, while at ER only one sequence type was recorded. In the case of *O. atlantica*, BB showed the highest sequence diversity value, followed by MAB, while at RdP, 7AR and 8AR, no sequence diversity was detected, since only one sequence type was found (Table 1).

**Figure 2:**
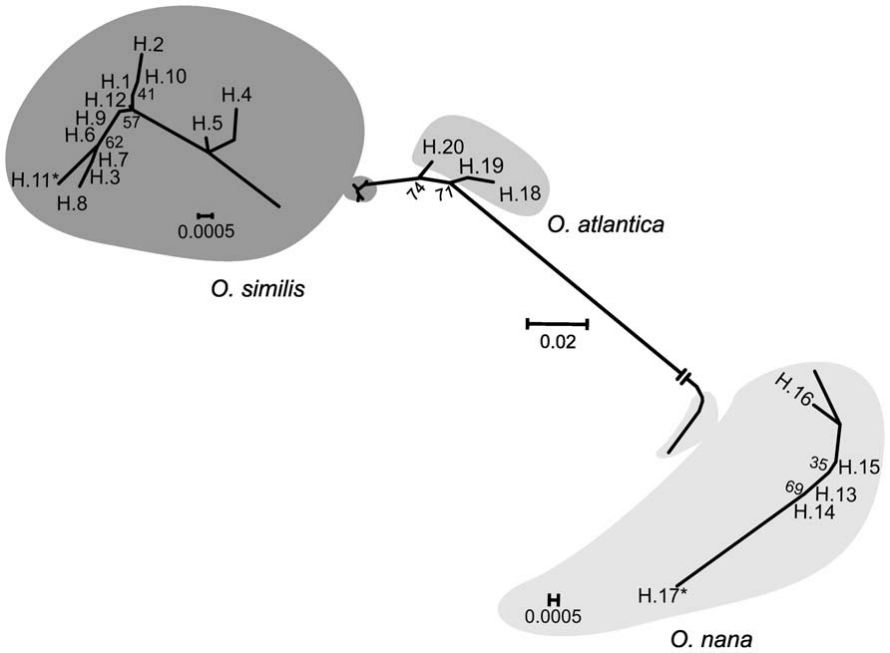
Relationships among the three *Oithona* species based on 28S rDNA. Unrooted Neighbor-Joining analysis under the Jukes-Cantor model, showing relationships among the three *Oithona* species based on 28S rDNA sequence types of *O. similis* (H1–H12), *O. atlantica* (H13–H17) and *O. nana* (H18–H20). Sequence types found at each species’ type locality are indicated by asterisk (*). Numbers in the nodes indicate the percentage bootstrap recovery after 10,000 repetitions.

The A.I.C. selected the Jukes-Cantor [54] with alpha parameter for the gamma distribution of 0.25 as the evolutionary model that best fit the observed sequence variation. Mean Jukes-Cantor distances within species ranged from 0.001 for *O. similis* to 0.015 for *O. atlantica* (Table 2). Genetic distance between species was highest between *O. nana* and the other two species, with *O. nana* differing from *O. similis* by a distance of 0.224 and from *O. atlantica* by 0.222; the distance between *O. similis* and *O. atlantica* was much lower at 0.034 (Figure 2, Table 2).

**Table 2:** Relationships among the three *Oithona* species based on 28S rDNA.

Species	*O. atlantica*	*O. similis*	*O. nana*
*O. atlantica*	0.015 (0.008)		
*O. similis*	0.034 (0.009)	0.001 (0.001)	
*O. nana*	0.222 (0.014)	0.244 (0.013)	0.006 (0.005)

Mean Jukes-Cantor distances within (diagonal) and between (below diagonal) the three *Oithona* species. Distances among sequence types were calculated with MEGA (Ver. 5.05 using the Jukes-Cantor model with alpha parameter of 0.25. The standard deviation about each mean is indicated in parentheses. Numbers of specimens used for the analysis are: *O. similis* (108), *O. atlantica* (23), and *O. nana* (19).

### 28S rDNA Variation of O. similis

**Figure 3:**
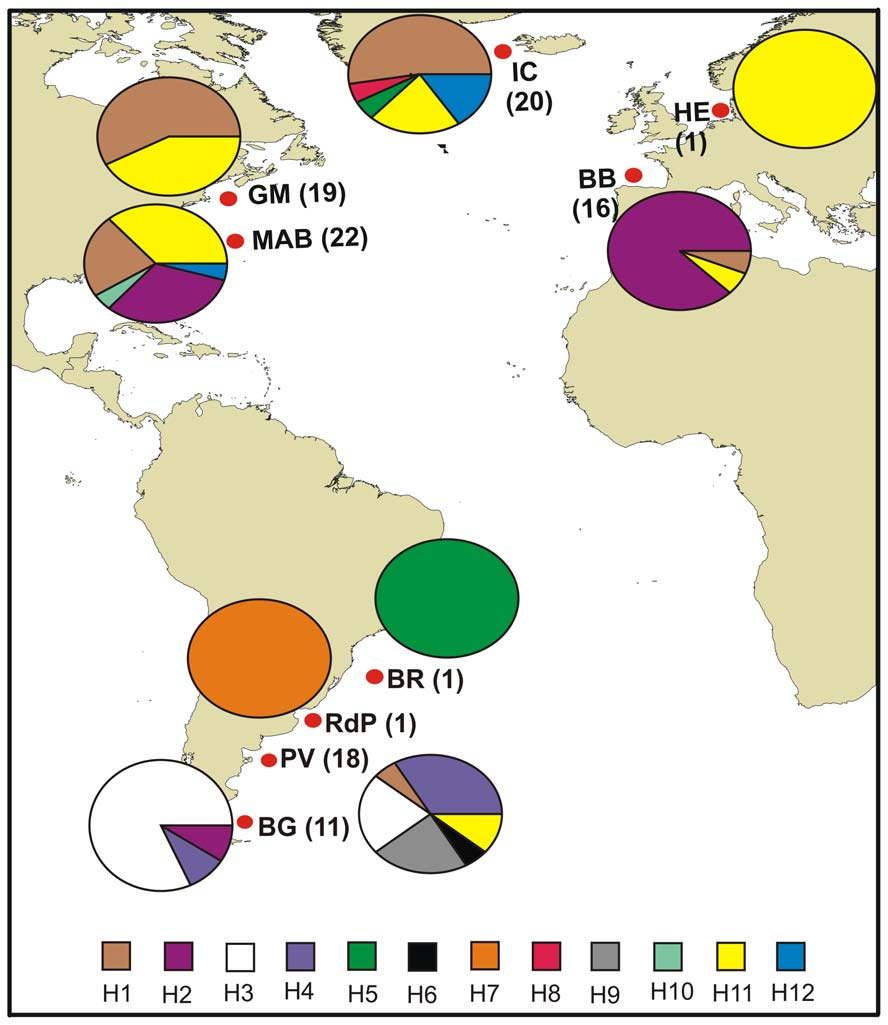
Distribution and frequence of *Oithona* similis kind sequence. Pie diagrams depicting the kind sequence frequencies of a 51****bp region of 28S for samples of *O. similis* collected from Gulf of Maine (GM), Iceland (IC), Middle Atlantic Bight (MAB), Bay of Biscay (BB), Península Valdés (PV) and Bahía Grande (BG). Sample size (n =  number of individual copepods) in each location. The twelve *O. similis* sequence types (H1–H12) are represented by different colours. References in the figure.

Among twelve 28S rDNA sequences detected for *O. similis,* H1, H2, H5 and H11 were present in both hemispheres (Figure 3). H1 was the most frequently found, distributed at GM, BB, IC, MAB and PV. H11 was found in GM, BB, IC, MAB, HE and PV, while H2 was present in BB, MAB, and BG. H5 was in IC and BR (Figure 3).

Three sequences were exclusively found in the Northern Hemisphere. They were only present at IC (H8, H12) and MAB (H10, H12) (Figure 3). Five sequences occurred only in the Southern Hemisphere: H3, H4, H6, H7 and H9 (Figure 3). The most frequently found were H3, H4 and H9 which were present at BG and PV. H6 and H7 were only found at PV and RdP (Figure 3). Φ_ST_ values [53] derived from genetic distances were significant for all pairwise comparisons between populations except for the pairs GM-IC and PV-BG. Thus, *O. similis* populations were tentatively separated into four groups: GM+IC; MAB; BB; and PV+BG (Table 3). This clustering was supported by AMOVA analysis, which revealed that 53.58% of the observed genetic variation was among groups, and 37.94% was within populations (Table 4).

**Table 3:** Pairwise Φ_ST_ distances between all *O. similis* populations with n>1.

	GM	IC	PV	BG	MAB	BB
GM	-					
IC	0.139	-				
PV	0.603**	0.528**	-			
BG	0.907**	0.856**	0.216	-		
MAB	0.419**	0.248*	0.286**	0.586**	-	
BB	0.886**	0.817**	0.504**	0.687**	0.405*	-

Asterisks indicate the significance level (p) for each comparison calculated from 10,000 permutations: p<0.001 (*); p<0.0001 (**). Numbers of specimens used for the analysis are: GM (19), BB (16), IC (20), MAB (21), PV (18) and BG (11).

**Table 4:** Analysis of MOlecular VAriance (AMOVA) based on 28S rDNA sequence data for *Oithona similis*.

Observed partition					
Source of variation	Variance	%	Φ-statistics	P-value	d.f.
Among groups	0.646	53.58	Φ_ST_ = 0.535	0.02	3
Among populations within groups	0.102	8.49	Φ_SC_ = 0.182	0.01	2
Within populations	0.457	37.94	Φ_ST_ = 0.620	< 0.01	102

Variance and percentage of variance explained (%), fixation indexes (Φ-statistics), P-value indicates probability of obtaining a higher Φ value by chance estimated by 10,000 permutations, d.f.: degrees of freedom. (Refer to Method text for the definition of group and population).

## Discussion

Accurate and reliable identification of species is a necessary foundation for assessment of biodiversity, especially for important but lesser-known regions of the world ocean, such as the Argentine Sea [55]. DNA sequence variation of target genes provides invaluable tools for such analyses.

This study examined variation of a portion of 28S (large subunit) rDNA as a marker to identify and discriminate species of the ecologically-important but understudied cyclopoid copepod genus *Oithona*, found in the Argentine Sea - Southwest Atlantic Ocean - and North Atlantic Ocean. The species analyzed here, *O. similis, O. nana* and *O. atlantica,* were confirmed by molecular analysis to be distinct species, as previously characterized by morphological taxonomic analysis [7], [8], [24], [25]. Inclusion in our analysis of *O. similis* and *O. nana* from the type localities was particularly useful to allow determination of reference sequences for these species for future comparisons.

The genetic distances observed within and between species of *Oithona* agreed somewhat with those reported by Ueda et al. [56]. Our distance values were higher than those registered by these authors; which could be related to the fact that they analyzed two size forms of *O. dissimilis*. Our interspecific genetic distances may reflect the relationships registered by Nishida [8].

In addition to characterizing differences between species, the present work provided preliminary analysis of the levels and patterns of 28S rDNA sequence variation within each of the studied species based on samples collected from a broad latitudinal range of the Atlantic Ocean. Shared kind sequences were detected between North and South Atlantic collections for each of the *Oithona* species analyzed, despite the large distances between sampling locations. This finding confirms that 28S rDNA serves as a useful genetic marker for identification of these – and likely all – *Oithona* species, even those with global distributions.

Levels of intraspecific variation differed among the species: DNA sequence variation (measured as the percentages of bases) was higher for *O. atlantica* (1.5%) than either *O. nana* (0.6%) or *O. similis* (0.1%). The lower values recorded for *O. nana* and *O. similis,* which are both found commonly in coastal and shelf waters, might be due in part to their introduction by ballast water. For the Argentine Sea, [57] reported the presence of *O. nana* and *O. similis* in ballast water from commercial vessels from several origins (*e.g.,* Indian and Pacific Ocean, Mediterranean and Baltic Seas and Atlantic ports north of 20°S). At the Russian port of Novorossiysk, high abundances (10,000 individuals/m^3^) of live individuals of *O. nana* were found in samples taken from ships’ ballast water [58]. Interestingly, *O. similis* exhibited significantly different genetic differences among populations sampled for this study, although these differences were the lowest of the three species examined were not correlated with geographic distances, since some samples differed markedly despite their geographic proximity (*e.g.,* GM and MAB).

Based on 28S rDNA, *O. similis* is a single, genetically-cohesive species throughout the studied distributional range. Even for this conserved genetic marker, the species showed significant genetic differentiation among regions of the North and South Atlantic Oceans. It seems likely that geographic populations of *O. similis* might be primarily isolated by large-scale patterns of ocean circulation, as has been suggested by other genetic analysis of zooplankton in the Atlantic Ocean basin [44], [59], [60].

Our analysis of intraspecific and interspecific patterns of variation for three species of *Oithona* in selected regions of the North and South Atlantic Oceans demonstrated the usefulness of the 28S rDNA as an accurate and reliable means of identifying and discriminating the species. The 28S rDNA fragment we focused on is included the D1–D2 region, and has been suggested by Sonnenberg et al. [61] as a taxonomic marker due to its variability. Previous studies have used this marker for analysis of copepods [62] and other taxa [63]. Additional analysis of intraspecific variation, including studies using more highly variable molecular markers, will be needed to addresss questions of population connectivity, barriers to genetic cohesion, and discovery of cryptic species among such globally-distributed taxa.

## Supporting Information

Figure S1 **Alignment of the twelve 28S rDNA kind sequences of** ***Oithona*** **species.**
(FASTA)

## References

[1] Dana JD (1853). Crustacea..

[2] Paffenhöfer GA (1993). On the ecology of marine cyclopoid copepods (Crustacea Copepoda Cyclopoida).. J Plankton Res.

[3] Gallienne CP, Robins DB (2001). Is *Oithona* the most important copepod in the world’s oceans?. J Plankton Res.

[4] Nielsen TG, Møller EF, Satapoomin S, Ringuette M, Hopcroft RR (2002). Egg hatching rate of the cyclopoid copepod *Oithona similis* in arctic and temperate waters.. Mar Ecol Prog Ser.

[5] Castellani C, Irigoien X, Harris RP, Holliday NP (2007). Regional and temporal variation of *Oithona* spp. biomass, stage structure and productivity in the Irminger Sea, North Atlantic.. J Plankton Res.

[6] Nishida S, Tanaka O, Omori M (1977). Cyclopoid copepods of the family Oithonidae in Suruga Bay and adjacent waters.. Bull Plankton Soc Japan.

[7] Shuvalov VS (1980). Copepod cyclopoids of the family Oithonidae of the World Ocean..

[8] Nishida S (1985). Taxonomy and distribution of the family Oithonidae (Copepoda: Cyclopoida) in the Pacific and Indian Oceans.. Bull Ocean Res Inst Univ Tokyo.

[9] Dvoretsky VG, Dvoretsky AG (2009). Morphological plasticity in the small copepod *Oithona similis* in the Barents and White Seas.. Mar Ecol Prog Ser.

[10] Fish CJ (1936). The biology of *Oithona similis* in the Gulf of Maine and Bay of Fundy.. Biological Bulletin, Marine Biological Laboratory, Woods Hole.

[11] Nielsen TG, Sabatini ME (1996). Role of cyclopoid copepods *Oithona* spp. in North Sea plankton communities.. Mar Ecol Prog Ser.

[12] Williams JA, Muxagata E (2006). The seasonal abundance and production of *Oithona nana* (Copepoda: Cyclopoida) in Southampton Water.. J Plankton Res.

[13] Claus C (1866). Die Copepoden-Fauna von Nizza. Ein Beitrag zur Charakteristik der Formen und deren Abänderungen “im Sinne Darwin’s”.. Schr Ges Beförd ges Naturw.

[14] Claus C (1863). Die freilebenden Copepoden mit besonderer Berücksichtigung der Fauna Deutslands, der Nordsee und des Mittelmeeres, Leipzig.

[15] Razouls C, de Bovée F, Kouwenberg J, Desreumaux N (2005-2010). Diversité et répartition géographique chez les Copépodes planctoniques marins.. http://copepodes.obs-banyuls.fr.

[16] Farran GP (1908). Second report on the Copepoda of the Irish Atlantic slope.. Fish Ireland Sci Inv.

[17] Giesbrecht W (1892). Systematik und Faunistik der pelagischen Copepoden des Golfes von Neapel.. Fauna Und Flora des Golfes von Neapel,.

[18] Mazzocchi MG, Zagami G, Ianora A, Guglielmo L, Ianora A (1995). Systematic Account.. Atlas of Marine Zooplancton Straits of Magellan.

[19] Berasategui A, Menu Marque S, Gómez Erache M, Ramírez F, Mianzan H (2006). Copepod assemblages in a highly complex hydrographic region.. Estuar Coast Shelf Sci.

[20] Sabatini ME (2008). El ecosistema de la plataforma patagónica austral, Marzo-Abril 2000. Composición, abundancia y distribución del zooplancton.. Rev Invest Desarr Pesq.

[21] Di Mauro R, Capitanio F, Viñas M (2009). Capture efficiency for small dominant mesozooplankters (Copepoda, Appendicularia) off Buenos Aires province (34°S–41°S), Argentina, using two plankton mesh sizes.. Braz J Oceanogr.

[22] Antacli JC, Hernández D, Sabatini ME (2010). Estimating copepods' abundance with paired nets: Implications of mesh size for population studies.. J Sea Res.

[23] Marques E (1966). Copépodes des eaux de Boma et de l'embouchure du fleuve Congo.. Revue Zool Bot Afr.

[24] Ramírez FC (1966). Copépodos ciclopoideos y harpacticoideos del plancton de Mar del Plata.. Physis.

[25] Ramírez FC (1971). Copépodos planctónicos de los sectores bonaerense y norpatagónico. Resultados de la Campaña Pesquería III. Revista del Museo de La Plata, n.. s Zoología.

[26] Mori T (1964). The pelagic copepods from the neighbouring waters of Japan..

[27] Viñas MD, Negri RM, Ramírez FC, Hernández D (2002). Zooplankton assemblages and hydrography in the spawning area of anchovy (*Engraulis anchoita*) off Rio de la Plata estuary (Argentina–Uruguay).. Mar Freshwater Res.

[28] Marrari M, Viñas MD, Martos P, Hernández D (2004). Spatial patterns of mesozooplankton distribution in the Southwestern Atlantic Ocean (34°S–41°S) during austral spring: relationship with the hydrographic conditions.. ICES J Mar Sci.

[29] Viñas MD, Ramírez FC (1996). Gut analysis of first-feeding anchovy larvae from the Patagonian spawning areas in relation to food availability.. Arch Fish Mar Res.

[30] Viñas MD, Santos BA (2000). First-feeding of hake (*Merluccius hubbsi*) larvae and prey availability in the North Patagonian spawning area. Comparison with anchovy.. Arch Fish Mar Res.

[31] Ferrari FD, Bowman TE (1980). Pelagic copepods of the family Oithonidae (Cyclopoida) from the east coasts of Central and South America.. Smithsonian Contributions to Zoology.

[32] Bradford-Grieve JM, Markhaseva EL, Rocha CEF, Abiahy B (1999). Copepoda. In Boltovskoy D, editors. South Atlantic Zooplakton. Vol. 2.. Backhuys Publishers, Leiden,.

[33] Früchtl F (1920). Plankton Copepoden aus der nordlichen Adria.. Sber Akad Wiss Wien Math Nat Kl.

[34] Ortman BD (2008). DNA barcoding the medusozoa and ctenophora..

[35] Struck TH, Schult N, Kusen T, Hickman E, Bleidorn C (2007). Annelid phylogeny and the status of Sipuncula and Echiura. BMC Evolutionary Biology 7:57.. http://www.biomedcentral.com/1471-2148/7/57.

[36] Bik HM, Lambshead PJD, Thomas WK, Lunt DH (2010). http://www.biomedcentral.com/1471-2148/10/353.

[37] Holznagel WE, Colgan DJ, Lydeard C (2010). Pulmonate phylogeny based on 28S rRNA gene sequences: A framework for discussing habitat transitions and character transformation.. Mol Phyl Evol.

[38] Borchiellini C, Chombard C, Manuel M, Alivon E, Vacelet J (2004). Molecular phylogeny of Demospongiae: implications for classification and scenarios of character evolution.. Mol Phyl Evol.

[39] Bucklin A, Harris RP, Weibe PH, Lenz J, Skjoldal HR, Huntley M (2000). Methods for population genetic analysis of zooplankton.. ICES Zooplankton Methodology Manual.

[40] White TJ, Bruns T, Lee S, Taylor J, MA InInnis, DH Gelfand, JJ Sninsky, TJ White (1990). Amplification and direct sequencing of fungal ribosomal RNA genes for phylogenetics.. editors.

[41] Folmer O, Black M, Hoeh W, Lutz R, Vrijenhoek R (1994). DNA primers for amplification of mitochondrial cytochrome c oxidase subunit I from diverse metazoan invertebrates.. Mol Mar Biol Biotechnol.

[42] Machida RJ, Miya MU, Nishida M, Nishida S (2002). Complete mitochondrial DNA sequence of *Tigriopus japonicus* (Crustacea: Copepoda).. Mar Biotech.

[43] Voznesenskya M, Lenz PH, Spanings-Pierrot C, Towle DW (2004). Genomic approaches to detecting thermal stress in *Calanus finmarchicus* (Copepoda: Calanoida).. J Exp Mar Biol Ecol.

[44] Unal E, Bucklin A (2010). Basin-scale population genetic structure of the planktonic copepod *Calanus finmarchicus* in the North Atlantic Ocean.. Progr Oceanogr.

[45] Thompson JD, Higgins DG, Gibson JJ (1994). Clustal W: improving the sensitivity of progressive multiple sequence alignment through sequence weighting, position specific gap penalties and weight matrix choice.. Nucleic Acids Res.

[46] Tamura K, Peterson D, Peterson N, Stecher G, Nei M (2011). MEGA5: Molecular Evolutionary Genetics Analysis using Maximum Likelihood, Evolutionary Distance, and Maximum Parsimony Methods.. Mol Biol Evol.

[47] Librado P, Rozas J (2009). DnaSP v5: a software for comprehensive analysis of DNA polymorphism data.. Bioinformatics.

[48] Petit JR, El Mousadik A, Pons O (1998). Identifying populations for conservation on the basis of genetic markers.. Cons Biol.

[49] Posada D (2008). jModelTest: Phylogenetic Model Averaging.. Mol Biol Evol.

[50] Saitou N, Nei M (1987). The neighbor-joining method: A new method for reconstructing phylogenetic trees.. Mol Biol Evol.

[51] Felsenstein J (1985). Confidence limits on phylogenies: An approach using the bootstrap.. Evolution.

[52] Excoffier L, Lischer HL (2010). Arlequin suite ver 3.5: a new series of programs to perform population genetics analyses under Linux and Windows.. Mol Ecol Resour.

[53] Excoffier L, Smouse PE, Quattro JM (1992). Analysis of molecular variance inferred from metric distances among DNA haplotypes: application to human mitochondrial DNA restriction data.. Genetics.

[54] Jukes TH, Cantor CR (1969). Evolution of Protein Molecules..

[56] Ueda H, Yamaguchi A, Saito S, Sakaguchi S, Tachihara K (2011). Speciation of two salinity-associated size forms of *Oithona dissimilis* (Copepoda: Cyclopoida) in estuaries.. J Nat Hist.

[57] Boltovskoy D, Almada P, Correa N (2011). Biological invasions: assessment of threat from ballast-water discharge in Patagonian (Argentina) ports.. Environ Sci Pol.

[58] Selifonova, ZhP (2009). *Oithona brevicornis* Giesbrecht (Copepoda, Cyclopoida) in harborages of the northeastern part of the Black Sea shelf.. Inland Water Biol.

[59] Bucklin A, Astthorsson OS, Gislason A, Allen LD, Smolenack SB (2000). Population genetic variation of *Calanus finmarchicus* in Icelandic waters: preliminary evidence of genetic differences between Atlantic and Polar populations.. ICES J Mar Sci.

[60] Goetze E (2003). Cryptic speciation on the high seas; global phylogenetics of the copepod family Eucalanidae.. Proc R Soc Lond B.

[61] Sonnenberg R, Nolte A, Tautz D (2007). An evaluation of LSU rDNA D1-D2 sequences for their use in species identification. Front Zool 4, 6.. http://www.frontiersinzoology.com/content/4/1/6.

[62] Hayward CJ, Svane I, Lachimpadi SK, Itoh N, Bott N, J (2011). Sea lice infections of wild fishes near ranched southern bluefin tuna (*Thunnus maccoyii*) in South Australia.. Aquaculture.

[63] Brown L, Bresnan E, Graham J, Lacaze JP, Turrell E (2010). Distribution, diversity and toxin composition of the genus Alexandrium (Dinophyceae) in Scottish waters. Eur.. J Phycol.

